# Decoding Cellular Communication Networks and Signaling Pathways in Bone, Skeletal Muscle, and Bone-Muscle Crosstalk Through Spatial Transcriptomics

**DOI:** 10.21203/rs.3.rs-6701121/v1

**Published:** 2025-06-12

**Authors:** Hong-Wen Deng, Chuan Qiu, Yisu Li, Yun Gong, Weiqiang Lin, Boluwatife Afolabi, Vivek Thumbigere-Math, Kuan-Jui Su, Jeffrey Deng, Shashank Sajjan Mungasavalli Gnanesh, Zhe Luo, Qing Tian, Yiping Chen, Hui Shen

**Affiliations:** Tulane University; Tulane University; Tulane University; Tulane; Tulane University; Tulane University; University of Maryland Baltimore; Tulane University; Dartmouth College; Tulane University; Tulane University; Tulane University; Tulane University; Tulane University

**Keywords:** bone, skeletal muscle, spatial transcriptome, cell-cell communication

## Abstract

Bone and skeletal muscle are essential components of the musculoskeletal system, enabling movement, load-bearing, and systemic regulation. These tissues communicate through dynamic bone-muscle crosstalk mediated by cytokines, growth factors, and extracellular matrix (ECM) proteins. The spatial organization of these mediators is critical to maintaining tissue integrity, and disruptions contribute to diseases such as osteoporosis, sarcopenia, and metabolic syndrome. Despite the importance of spatial context, studies using spatial transcriptomics (ST) to investigate bone-muscle interactions remain limited.

Here, we applied 10X Genomics Visium ST profiling and advanced computational tools to characterize cell-cell communication networks and ligand-receptor (L-R) interactions in mouse femur and adjacent skeletal muscle. We identified eight major cell types: erythroid cells, endothelial cells, skeletal muscle cells, osteoblasts, myeloid cells, monocytes/macrophages, mesenchymal stem cells, and adipocytes, each exhibiting distinct spatial gene expression profiles.

Signaling pathway analysis revealed 13 key pathways mediating intra- and inter-tissue communication, including COLLAGEN, THBS, VEGF, FN1, and TENASCIN. Notable L-R pairs involved in bone, muscle, and bone-muscle crosstalk include Col1a2-Sdc4 (osteoblast-ECM interactions), Tnxb-Sdc4 (muscle-to-endothelial signaling), Vegfa-Vegfr1 and Vegfa-Vegfr2 (muscle-to-endothelial/myeloid signaling), and Comp-Sdc4 (monocyte/macrophage-to-osteoblast signaling).

This study presents the first spatially resolved map of cell-cell communication across bone and skeletal muscle, providing novel insights into their molecular crosstalk. These findings offer a critical foundation for future therapeutic strategies targeting musculoskeletal disorders.

## Introduction

Bone and skeletal muscle are integral components of the musculoskeletal system, functioning not only as structural elements essential for movement and load-bearing but also as key regulators of systemic homeostasis (1). The dynamic interplay between these tissues, commonly referred to as bone-muscle crosstalk, encompass a complex network of biochemical signals mediated by cytokines, growth factors, and extracellular matrix (ECM) proteins (2), alongside their mechanical interactions. This intricate communication is essential for maintaining tissue homeostasis, coordinating responses to mechanical stress, and facilitating repair following injury (3, 4). Dysregulation of bone, skeletal muscle, and their crosstalk has been implicated in the pathogenesis of musculoskeletal and metabolic disorders, such as osteoporosis, sarcopenia, and metabolic syndrome (3–8), highlighting the importance of bone-muscle interactions in both fundamental and translational research. Recently evidence underscores the molecular interdependence of bone and skeletal muscle (2, 9–14). For instance, bone-derived factors, such as osteocalcin, have been shown to influence muscle metabolism (11), while muscle-secreted myokines, such as irisin, regulate bone remodeling and systemic energy expenditure (12). Additionally, several key signaling pathways, such as COLLAGEN and SPP1, play pivotal roles in the physiology of both tissues (13, 14). The COLLAGEN signaling pathway is fundamental for providing tensile strength to bone and muscle, supporting ECM organization, and regulating osteoblast and osteoclast activity (13). Similarly, the SPP1 signaling pathway is essential for bone remodeling, hematopoietic stem cell niche maintenance, and muscle repair (14). These discoveries have reshaped our understanding of musculoskeletal biology and emphasize the necessity of integrative approaches to studying bone-muscle crosstalk.

Beyond biochemical and mechanical interactions, the spatial organization of bone and muscle plays a pivotal role in regulating their functions (6, 15–20). The distribution of biochemical signals, ECM composition, and cellular interactions varies across anatomical regions, influencing local tissue responses to mechanical load, injury, and aging (2–4). Recognizing the spatial diversity of these interactions is essential for developing targeted therapeutic strategies and refining experimental models that more accurately capture the complexity of musculoskeletal biology. Advancements in single-cell RNA sequencing (scRNA-seq) have significantly enhanced our understanding of cellular diversity and gene expression profiles in bone and muscle tissues (21–26). However, scRNA-seq has inherent limitations in capturing the spatial organization of cells, making it difficult to contextualize cellular changes within their native microenvironments or to map spatially dependent intercellular interactions. For instance, while scRNA-seq studies have identified key osteogenic and myogenic transcriptional programs, they have not delineated the spatial organization of these processes or their influence on neighboring cells (26, 27). Spatial transcriptomics (ST), an innovative technology that integrates spatial localization with transcriptional profiling, has recently emerged as a powerful tool to overcome these limitations (28). By generating high-resolution gene expression maps within intact tissues, ST enables researchers to explore how cellular behavior is influenced by tissue architecture and intercellular communication (6, 28–30). For example, a recent study combined RNA tomography and mass spectrometry imaging to map the spatial transcriptomic, metabolomic, and lipidomic organization of the murine tibialis anterior muscle, revealing strong regionalization of gene expression, metabolic variation, and myofiber type distribution along the proximal-distal axis. Another study integrated ST and scRNA-seq to map skeletal stem and progenitor cells (SSPCs) within their native bone marrow niche. By leveraging multiple deconvolution methods, they successfully localized SSPC subtypes, identified key signaling networks, and uncovered spatially restricted metabolic and morphogenetic gradients. However, these studies primarily focus on skeletal muscle or bone tissue alone and lack a comprehensive and systematic analysis of cell-cell communication across diverse cell types and signaling pathways. Consequently, the application of ST in elucidating bone, skeletal muscle, and their crosstalk remains to be done. Further spatial transcriptomic profiling of both bone and skeletal muscle is essential to advancing our understanding of the regional specialization of bone-muscle interactions. Such studies will help identify unique molecular signatures that drive context-dependent physiological and pathological processes, ultimately deepening insights into musculoskeletal biology and potential therapeutic targets.

In this study, we performed spatial transcriptomic analyses of mouse femur bone and adjacent skeletal muscle tissues, which represent a critical interface for bone-muscle crosstalk and are subject to significant physiological and mechanical stress (31). By integrating ST with novel computational deconvolution algorism (32) and cell-cell communication analysis (33), we delineated the spatial transcriptional landscape of these tissues and identified several key ligand-receptor (L-R) interactions mediating their crosstalk through well-characterized signaling pathways. This approach overcame the spatial resolution limitations of scRNA-seq, providing innovative insights into the spatial organization and regulation of intercellular communication within the bone and skeletal muscle microenvironments. Our study establishes a comprehensive map of spatially resolved cell-cell communication networks in bone and skeletal muscle, revealing critical signaling pathways and L-R pairs that are fundamental to musculoskeletal health. These findings advance our understanding of the molecular mechanisms underlying bone, skeletal muscle and their crosstalk, as well as provide a robust framework for developing therapeutic strategies to restore tissue homeostasis and mitigate musculoskeletal and metabolic disorders.

## Results

### Spatial Transcriptional Profiling of Bone and Skeletal Muscle

**Figure 1:**
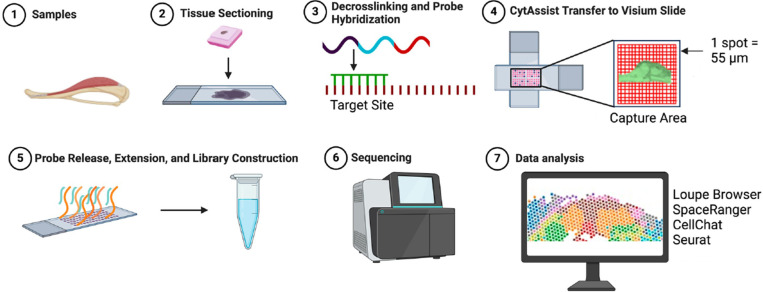
Overview of 10X Genomics CytAssist Visium ST platform. (1) Mouse femur is harvested at two weeks. (2) Femur is fixed with 4% paraformaldehyde and embedded in paraffin. Tissue is sectioned at 4 μm and then one slide was used for H&E staining and following ST analysis. (3) Decrosslinking and probe hybridization are performed. (4) The user supplied slide is then aligned with a Visium CytAssist slide using a 6.5 mm by 6.5 mm capture area for sequencing. The capture area contains ~5,000 tissue capture spots (each spot is 55 μm in diameter). (5) Attached probes are released with RNAse allowing for probe extension and subsequent library construction. Each probe is now attached with a barcoded label with spatial information. (6) Sequencing is performed. (7) The final step is data analysis by the user. Popular bioinformatics options include Loupe Browser (10X Genomics), SpaceRanger (10X Genomics), Seurat, and CellChat etc.

**Figure 2:**
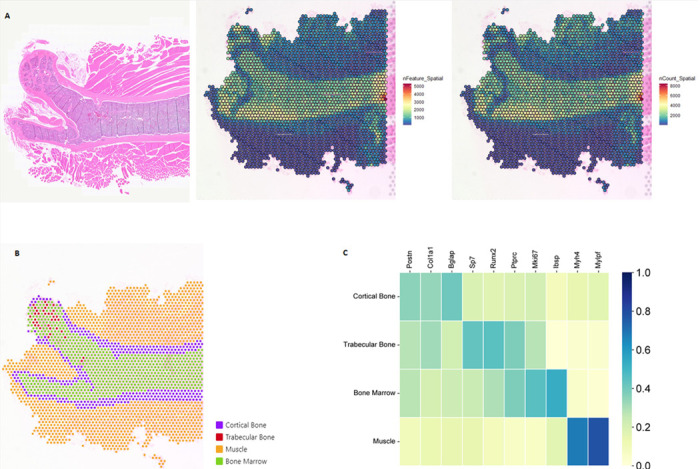
Spatial transcriptional profiling of femur and surround skeletal muscle. **A)** Spatial feature plots of H&E-stained sections overlaid with the number of unique genes (nFeature) or unique transcripts (nCount) per spatial spot. **B)** Manual segmentation of cortical bone, trabecular bone, bone marrow, and muscle spatial spots by histological morphology. **C)**Marker genes showing enriched expression within each segmented compartment.

To address the challenges of spatially resolving transcriptional landscapes in bone and skeletal muscle tissue, we employed 10X Genomics Visium ST to analyze femur and adjacent skeletal muscle tissues from a male C57BL/6 mouse. (Fig. 1). Our quality control metrics identified 2,660 spatial spots, with a median of 506 unique genes per spot, encompassing a total of 19,199 unique genes (Table 1, Fig. 2A). Notably, the number of unique genes per spot identified in our study exceeds that reported in previous study (6), highlighting the high quality and robustness of our dataset. The high efficiency of transcript recovery underscored the robustness of our workflow, particularly for challenging tissues such as bone. To assess tissue-specific gene expression changes and validate our findings against previous results (6), we manually segmented the spatial data into cortical bone (purple), trabecular bone (red), bone marrow (green), and muscle (yellow) regions based on H&E imaging (Fig. 2B). This segmentation allowed for precise comparisons and enhanced the spatial resolution of gene expression patterns across distinct musculoskeletal compartments. Notably, spatial spots were assigned to the tissue type that occupied the majority of the area within each spot. Consequently, some spots assigned to a specific tissue (e.g., “cortical bone”) may also contain cells from adjacent regions due to partial overlap (e.g., cortical bone spots overlapping with bone marrow). As shown in Fig. 2C, gene expression profiles confirmed successful spatial delineation of captured transcripts. Both cortical and trabecular bone regions exhibited high expression levels of mature osteogenic transcripts such as *Col1a1.* Additional osteogenic markers, including mature osteogenic/periosteal transcripts *(Bglap/Postn)* and osteoprogenitor genes *(Sp7, Runx2), were* enriched in cortical and trabecular bone, respectively. In contrast, hematopoietic markers *(Ptprc)* and proliferative markers *(Mki67, Ibsp)* were predominantly enriched within the bone marrow, consistent with gene expression pattern identified in previous findings (6). Notably, we observed elevated expression of myosin genes *(Myh4, Mylpf)* in skeletal muscle tissue. These findings demonstrate, for the first time, the technical feasibility of conducting spatial transcriptomic analyses within mouse bone and adjacent skeletal muscle tissue, providing a new framework for understanding the spatially resolved transcriptional landscape and their communication of these tissues.

### Computational Deconvolution of Spatial Spots

The high complexity of bone and muscle tissues, characterized by the diversity of cell types in close proximity and their intricate interactions, poses a significant challenge for accurately assigning spatial gene expression profiles to individual cell types. This complexity is further exacerbated by the limited spatial resolution of current spatial transcriptomic technologies, which capture gene expression data as aggregates within spatial spots, lacking a single-cell resolution. To address these challenges, we employed SMART (32), which integrates spatial transcriptomic data with curated cell-type-specific marker genes to simultaneously infer cell type-specific gene expression profiles and estimate the cellular composition within each spatial spot. Marker genes were curated from the Mouse Cell Atlas (34) and the CellMarker 2.0 database (35), ensuring comprehensive and biologically relevant reference datasets.

**Figure 3:**
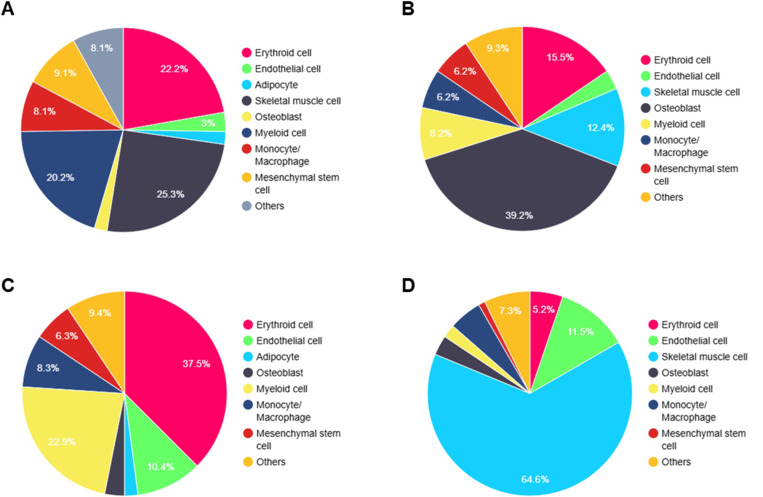
SMART-Predicted Cell Type Composition. **A)** Overall cell type composition across the femur and adjacent skeletal muscle tissue. **B)** Cell type composition specifically within bone regions. **C)** Cell type composition in the bone marrow microenvironment. **D)** Cell type composition in the skeletal muscle tissue.

Using SMART, we deconvolved the spatial transcriptomic dataset obtained from mouse bone and skeletal muscle tissues into eight major cell types, each comprising more than 2% of the total cellular composition. These cell types included erythroid cells, endothelial cells, adipocytes, skeletal muscle cells, osteoblasts, myeloid cells, monocytes/macrophages, and mesenchymal stem cells (Fig. 3A). This deconvolution allowed us to disentangle the mixed gene expression profiles at each spatial spot, accurately attributing transcriptional activity to specific cell types. As expected, we observed that osteoblast cluster were predominant in cortical and trabecular bone regions, reflecting their critical role in bone remodeling and ECM production. Mesenchymal stem cells were also enriched in these regions, suggesting their contribution to osteogenic progenitor activity (Fig. 3B). Hematopoietic cells, including erythroid and myeloid cells, dominated the bone marrow microenvironment, consistent with its function as a hematopoietic niche. Endothelial cells were also prominent, highlighting their role in vascular niches (Fig. 3C). Skeletal muscle cells were confined to the muscle regions, with minor infiltration of immune cells such as monocytes/macrophages, particularly at the muscle-bone interface (Fig. 3D). Adipocytes were sparsely distributed, aligning with their supportive role in marrow and stromal microenvironments. Next, we conducted a differential expression analysis to compare gene expression profiles between bone and skeletal muscle tissues. The differentially expressed genes identified were subsequently used for functional enrichment analysis. Notably, we observed significant enrichment in Gene Ontology (GO) terms related to muscle contraction and collagen metabolism (Table 2), reflecting the distinct functional roles of these tissues. The enrichment of muscle contraction-related GO terms aligns with the physiological role of skeletal muscle in generating force and movement, while the collagen metabolism-related GO terms highlight the importance of ECM remodeling in bone structure and function. In summary, these results revealed the intricate cellular architecture of bone and skeletal muscle tissues, providing a foundational framework for further exploration of cell-cell communication and functional dynamics within these interconnected systems.

### Cell-Cell Communication Networks in Bone and Skeletal Muscle

**Figure 4:**
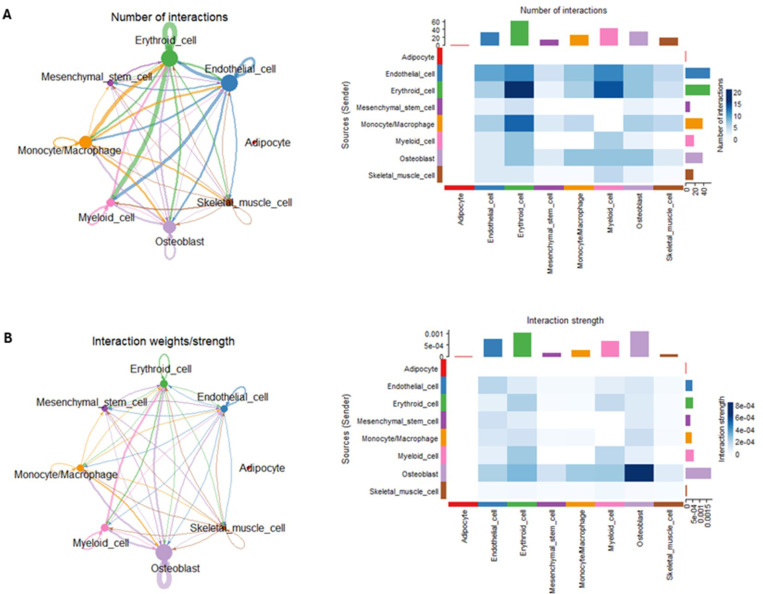
Crosstalk Pathways Within the Femur. **A)** Chord diagrams showing the number of calculated inter-cluster interactions within femur (left panel). Heatmap showing the number of interactions between clusters within femur (right panel). Color indicates increased number between the sending cluster and the receiving cluster. **B)** Chord diagrams showing the weight/strength of calculated inter-cluster interactions within femur (left panel). Heatmap showing the strength (right panel) of interactions between clusters within femur (right panel). Color indicates increased strength between the sending cluster and the receiving cluster.

Bone-muscle crosstalk has been extensively studied in both humans and mice, revealing its critical roles in maintaining musculoskeletal health, regulating systemic metabolism, and facilitating responses to mechanical stress and injury (2, 9–12). Emerging evidence underscores the complexity of communication processes between bone and muscle, highlighting the need to elucidate these interactions to enhance our understanding of systemic physiological regulation and the development of musculoskeletal and metabolic disorders (3, 36–38). Despite significant progress, spatially resolved transcript-level studies of bone-muscle interactions remain underexplored, leaving a critical gap in understanding the spatial organization and regulation of these interactions within the tissue microenvironment. To address this gap, we employed CellChat (35), a computational tool designed to infer intercellular communication networks by analyzing known L-R interactions and their expression patterns. By integrating spatially resolved data, CellChat incorporates physical proximity between tissue sites, enabling exclusion of unlikely interactions due to spatial separation. This approach provides a robust framework for mapping intercellular communication networks with high spatial precision. Our analysis revealed extensive intercellular communication networks within femur and skeletal muscle tissue (Fig. 4A-B). Among the cell populations, erythroid cells, endothelial cells, and monocytes/macrophages exhibited the highest levels of communication (>10 interactions), forming dense networks with most other cell populations (Fig. 4A). For example, the strongest interactions were observed between endothelial cells and erythroid/myeloid cells, erythroid and myeloid cells, as well as monocytes/macrophages and erythroid cells. Intriguingly, erythroid cells demonstrated strong autocrine signaling within their own population. Osteoblasts, while displaying a moderate number of intercellular interactions, showed the most robust communication with all other cell populations, including strong autocrine signaling (Fig. 4B). Skeletal muscle cells showed a moderate number of intercellular interactions with endothelial cells, erythroid, monocytes/macrophages, and osteoblasts. Conversely, adipocytes exhibited minimal detectable communication networks, indicating a limited role in the observed crosstalk within femur and skeletal muscle tissue.

**Figure 5:**
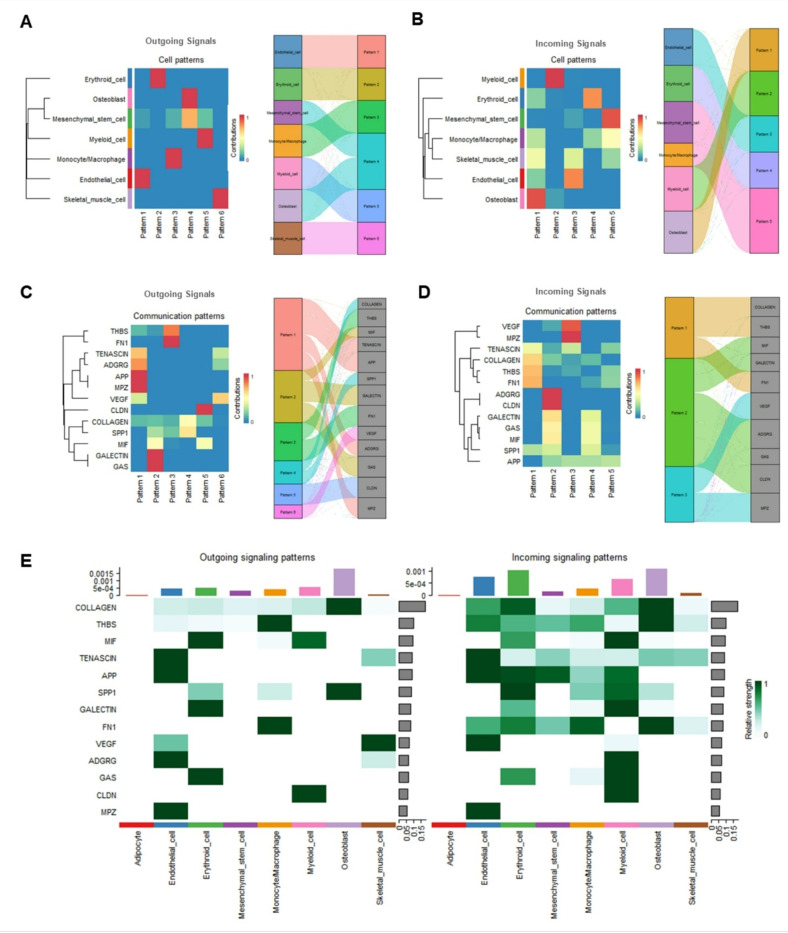
Heatmap showing cell signaling patterns within femur tissue. **A)** Outgoing cell signaling patterns in each cell cluster (left) and outgoing cell signaling patterns with cell clusters having contribution >0.5 (right). **B)** Incoming cell signaling patterns in each cell cluster (left) and incoming cell signaling patterns with cell clusters having contribution >0.5 (right). **C)** Outgoing pathway signaling patterns (left) and outgoing pathway signaling patterns with pathway having contribution >0.5 (right). **B)** Incoming pathway signaling patterns (left) and incoming pathway signaling patterns with pathway having contribution >0.5 (right). Color indicates how strongly a given cluster aligns with a specific pattern.

Next, we characterized both outgoing and incoming cell signaling patterns in femur and skeletal muscle tissue. As shown in Fig. 5, 6 distinct outgoing signaling patterns and 5 distinct incoming signaling patterns were identified by analyzing L-R interactions using probabilistic modeling and non-negative matrix factorization, offering insights into the functional organization of intercellular communication networks. For the outgoing signals, patterns 1,3, 4, and 5 were strongly associated with endothelial cells, monocytes/macrophages, osteoblasts, and myeloid cells, respectively. Notably, pattern 4 also demonstrated significant involvement of mesenchymal stem cells. In contrast, patterns 2 and 6 were predominantly restricted to erythroid cells and skeletal muscle cells, respectively (Fig. 5A). For the incoming signals, each pattern was shared across multiple cell clusters, indicating a more intricate and interconnected communication network compared to outgoing signaling patterns (Fig. 5B). To further elucidate the molecular mechanisms underlying these communication patterns, we performed pathway enrichment analysis and identified 13 key signaling pathways contributing to outgoing and incoming signals in femur and skeletal muscle tissue. These signaling pathways include COLLAGEN, THBS, MIF, TENASCIN, APP, SPP1, GALECTIN, FN1, VEGF, ADGRG, GAS, CLDN, and MPZ pathways. Each pathway was enriched in distinct communication patterns (Fig. 5C-D). For the outgoing signals, specific signaling pathways exhibited strong contributions (contribution > 0.5) to their corresponding communication patterns. TENASCIN, ADGRG, APP, and MPZ signaling pathways contributed strongly to pattern 1. GALECTIN, GAS, and MIF signaling pathways were dominant in pattern 2. THBS and FN1 signaling pathways played critical roles in pattern 3. COLLAGEN and SPP1 signaling pathways were key in pattern 4. CLDN and VEGF signaling pathways were associated with patterns 5 and 6, respectively (Fig. 5C). Similarly, for the incoming signals, COLLAGEN and THBS signaling pathways strongly contributed (contribution > 0.5) to pattern 1. ADGRG, CLDN, GALECTIN, GAS, and MIF signaling pathways were enriched in pattern 2. VEGF and MPZ signaling pathways dominated pattern 3 (Fig. 5D). The contribution of these signaling pathways to intercellular communication in various cell clusters were illustrated in Fig. 5E. For example, both outgoing and incoming signals in osteoblasts were mediated by COLLAGEN and SPP1 signaling pathways which play key roles in orchestrating bone matrix production, mineralization, and the structural integrity of bone tissue (13, 14), with additional contributions from THBS, FN1, and TENASCIN for incoming signals. Outgoing signals in skeletal muscle cells were driven by VEGF and TENASCIN signaling pathways which regulate angiogenesis, vascular niche maintenance, extracellular matrix (ECM) remodeling, and muscle repair, playing essential roles in bone-muscle crosstalk and facilitating tissue adaptation to mechanical stress (39–44), while VEGF was not involved in their incoming signaling networks. Monocytes/macrophages exhibited strong outgoing signals via THBS and FN1 signaling pathways, with osteoblasts being dominant receivers (Figs. 5E). This comprehensive mapping of signaling patterns and pathways highlights the complex interplay of cell-cell communication and key functional pathways, such as COLLAGEN, THBS, FN1, SPP1, VEGF, and TENASCIN, in femur and skeletal muscle tissue, offering valuable insights into the underlying molecular mechanisms driving bone-muscle crosstalk.

### Identification of L-R Interactions Among Cell Clusters Playing Key Roles in Bone and Skeletal Muscle

L-R interactions among cell clusters in bone and skeletal muscle are essential for maintaining tissue homeostasis, systemic metabolism, and coordinated responses to mechanical stress and injury (45–47). These interactions mediate bone-muscle crosstalk, regulate hematopoietic niches, and modulate immune signaling, thereby playing pivotal roles in musculoskeletal health (2, 9–14). Dysregulation of these signaling pathways is implicated in diseases, such as osteoporosis, sarcopenia, and metabolic disorders, highlighting their therapeutic potential (3–8). In this study, we investigated L-R interactions across several critical signaling pathways: COLLAGEN and SPP1 in osteoblasts; THBS and FN1 in monocytes/macrophages; and VEGF and TENASCIN in skeletal muscle cells. These signaling pathways govern bone and skeletal muscle metabolism, as well as regulate intercellular communication, providing a comprehensive map of cell-cell interactions in musculoskeletal tissues.

### The Role of L-R Interactions in COLLAGEN and SPP1 Signaling Pathways Regulating Osteoblast Function

**Figure 6:**
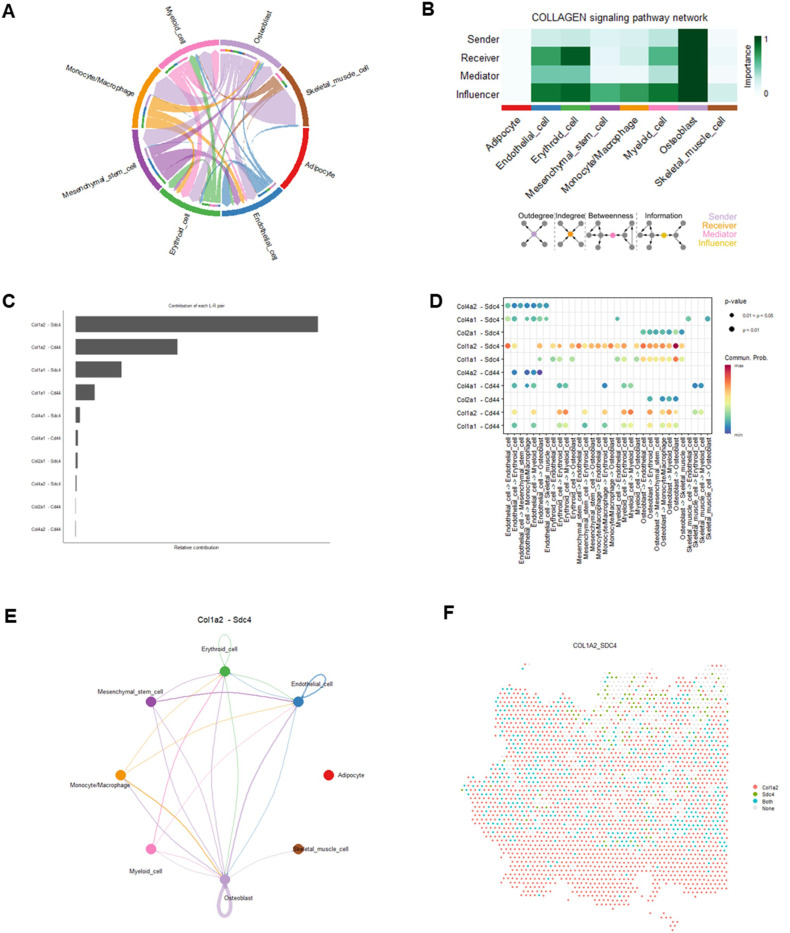
Cell-cell communications and their corresponding ligand-receptor interaction pairs in COLLAGEN pathway. A) A chord diagram shows the cell-cell communications in COLLAGEN pathway. B) The relative importance of each cell cluster based on the computed network centrality measures of signaling networks. Influencer represents a kind of cell that can control information flow within a signaling network, and a higher value indicates greater control on the information flow. The meaning of importance is the magnitude of the possibility of four roles (sender, receiver, mediator, and influencer) that the cell types play. The darker the color, the greater the role cells play. C) Relative contributions of each L-R pair. D) Dot plot shows the significant L-R pairs across various cell types in COLLAGEN signaling pathway. E) Cell-cell communication mediated by a Col1a2-Sdc4 L-R pair in COLLAGEN signaling pathway. F) A diagram shows the spatial localization of cell-cell communications via Col1a2-Sdc4 L-R pair in COLLAGEN pathway.

Osteoblasts emerged as central players in the COLLAGEN and SPP1 signaling pathways, which regulate ECM remodeling, cell adhesion, bone remodeling, and hematopoietic niches (48–52). Their multifaceted roles in these pathways highlight their importance in maintaining musculoskeletal homeostasis and mediating bone-muscle crosstalk (2, 53–55). In the COLLAGEN signaling pathway, we revealed a sophisticated network of cell-cell interactions (Figs. 6A). Most cell clusters exhibited high activity in the COLLAGEN signaling pathway as receivers and influencers. Notably, osteoblasts acted as dominant senders, receivers, mediators, and influencers, highlighting their critical role in the COLLAGEN signaling pathway (Fig. 6B). We identified 10 significant L-R pairs, encompassing 5 ligand genes (Col1a2, Collal, Col2a1, Col4a1, and Col4a2) and 2 receptor genes (Sdc4 and Cd44) (Figs. 6C-D, Supplemental Fig. 1). Among these L-R pairs, the Col1a2-Sdc4 pair emerged as the top contributor, mediating extensive interactions across cell clusters. These included outgoing signals from osteoblasts to most cell clusters, as well as outgoing signals from mesenchymal stem cells, monocytes/macrophages, and myeloid cells to endothelial cells, erythroid cells, and osteoblasts. Additionally, autocrine signaling within osteoblasts further highlighted the critical role of the Col1a2-Sdc4 L-R pair through COLLAGEN signaling pathway, contributing to ECM organization and bone remodeling (Fig. 6E-F). The SPP1 signaling pathway demonstrated a moderately complex interaction network (Supplemental Fig. 2A). Erythroid cells and monocytes/macrophages exhibited high activity in this pathway, functioning as receivers, mediators, and influencers, while osteoblasts emerged as dominant senders and influencers (Supplemental Fig. 2B). Osteoblasts interacted with erythroid cells, monocytes/macrophages and myeloid cells through a single L-R pair, Spp1-Cd44 (Supplemental Figs. 2C-D). Additionally, erythroid cells and monocytes/macrophages generated outgoing signals targeting myeloid cells and erythroid cells, respectively. Notably, robust autocrine signaling was observed in osteoblasts, monocytes/macrophages, and erythroid cells, emphasizing their regulatory roles through the Spp1-Cd44 L-R pair in the SPP1 signaling pathway (Supplemental Figs. 2CD).

### The Role of L-R Interactions in THBS and FN1 Signaling Pathways Regulating Monocytes/macrophages Function

**Figure 7:**
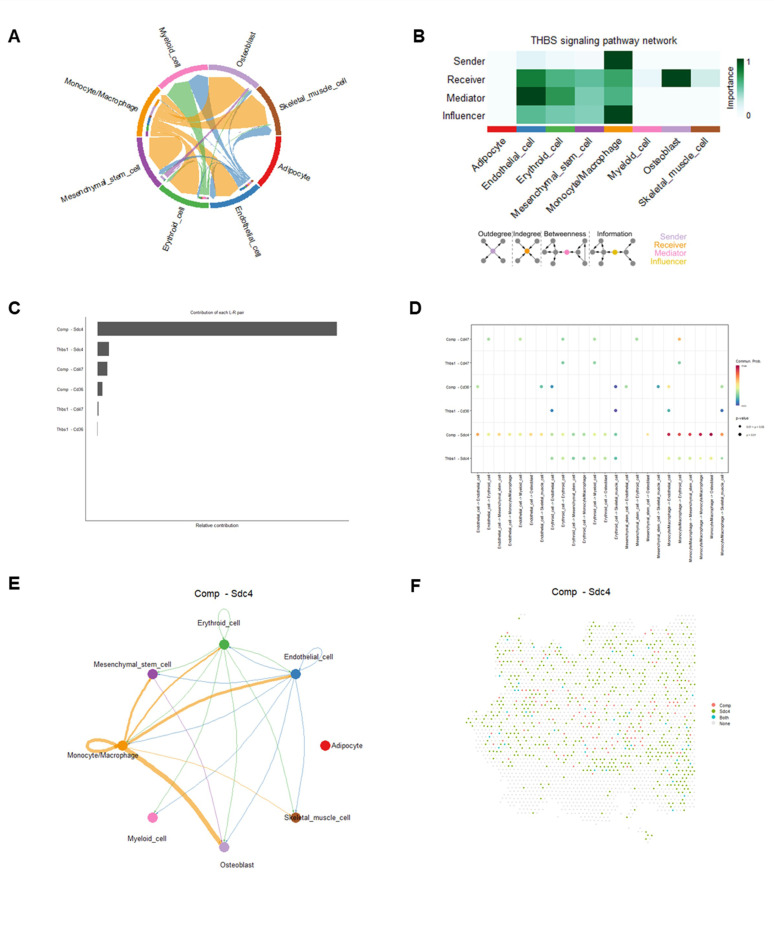
Cell-cell communications and their corresponding ligand-receptor interaction pairs in THBS pathway. A) A chord diagram shows the cell-cell communications in THBS pathway. B) The relative importance of each cell cluster based on the computed network centrality measures of signaling networks. Influencer represents a kind of cell that can control information flow within a signaling network, and a higher value indicates greater control on the information flow. The meaning of importance is the magnitude of the possibility of four roles (sender, receiver, mediator, and influencer) that the cell types play. The darker the color, the greater the role cells play. C) Relative contributions of each L-R pair. D) Dot plot shows the significant L-R pairs across various cell types in THBS signaling pathway. E) Cell-cell communication mediated by a Comp-Sdc4 L-R pair in THBS signaling pathway. F) A diagram shows the spatial localization of cell-cell communications via Comp-Sdc4 L-R pair in THBS pathway.

Monocytes/macrophages play a central role in the THBS and FN1 pathways, which regulate bone remodeling, ECM organization, stem cell niches, and muscle repair. Dysregulation in these pathways is linked to conditions such as osteoporosis, sarcopenia, and fibrosis, emphasizing their therapeutic importance (56–62). A highly interconnected network was identified within the THBS signaling pathway (Figs. 7A). Most cell clusters demonstrated high activity in the THBS signaling pathway as receivers, mediator, and influencers. Notably, monocytes/macrophages emerged as dominant senders, receivers, and influencers, whereas osteoblasts were primary receivers (Fig. 7B). 6 significant L-R pairs were identified, including 2 ligand genes (Thbs1 and Comp) and 3 receptor genes (Cd47, Cd36, and Sdc4), in the THBS signaling pathway (Figs. 7C-D, Supplemental Fig. 3). Notably, the Comp-Sdc4 L-R pair was a dominant contributor to cell-cell interactions, facilitating robust outgoing signals from monocytes/macrophages to osteoblasts, endothelial cells, mesenchymal stem cells, and skeletal muscle cells. Interesting, we also observed outgoing signals from mesenchymal stem cell, erythroid cells, and endothelial cells to osteoblast, as well as outgoing signals from erythroid cells and endothelial cells to skeletal muscle cells (Figs. 7E-F). In the FN1 signaling pathway, a sparse network of cell-cell interactions was observed (Supplemental Fig. 4A). Most cell clusters demonstrated high activity within the FN1 signaling pathway as receivers and influencers. Monocytes/macrophages notably emerged as dominant senders, receiver, and influencers, while osteoblasts primarily acted as receivers (Supplemental Fig. 4B). We identified 2 significant L-R pairs (Fn1-Sdc4 and Fn1-Cd44), mediating robust outgoing signals from monocytes/macrophages exhibited to osteoblasts, endothelial cells, mesenchymal stem cells, erythroid cells, and skeletal muscle cells, in the THBS signaling pathway (Supplemental Fig. 4C-D, Supplemental Fig. 5). Additionally, monocytes/macrophages exhibited strong autocrine signaling, highlighting their multifunctional roles in both the THBS and FN1 signaling pathways.

### The Role of L-R Interactions in VEGF and TENASCIN Signaling Pathways Regulating Skeletal Muscle Cells

**Figure 8:**
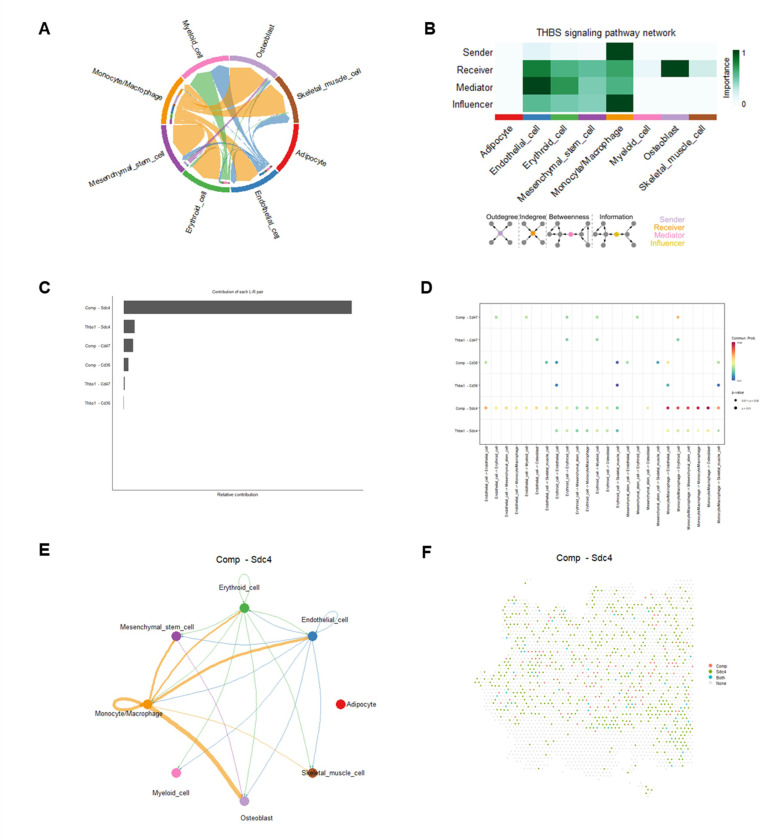
Cell-cell communications and their corresponding ligand-receptor interaction pairs in VEGF pathway. A) A chord diagram shows the cell-cell communications in VEGF pathway. B) The relative importance of each cell cluster based on the computed network centrality measures of signaling networks. Influencer represents a kind of cell that can control information flow within a signaling network, and a higher value indicates greater control on the information flow. The meaning of importance is the magnitude of the possibility of four roles (sender, receiver, mediator, and influencer) that the cell types play. The darker the color, the greater the role cells play. C) Relative contributions of each L-R pair. D) Dot plot shows the significant L-R pairs across various cell types in VEGF signaling pathway. E) Cell-cell communication mediated by a Vegfa-Vegfr1 L-R pair in VEGF signaling pathway. F) A diagram shows the spatial localization of cell-cell communications via Vegfa-Vegfr1 L-R pair in VEGF pathway.

Skeletal muscle cells actively participated in the VEGF and TENASCIN signaling pathways, which regulate angiogenesis, vascular niches maintenance, ECM remodeling, and muscle repair (41, 63, 64). These pathways play crucial roles in bone-muscle crosstalk and the adaptation of tissues to mechanical stress (39–44). The VEGF signaling pathway exhibited a sparse network of cell-cell interactions. Skeletal muscle cells demonstrated high activity within this pathway as senders and influencers. Endothelial cells, on the other hand, emerged as dominant receiver and influencers, reflecting their pivotal role in angiogenesis and vascular niche maintenance (Fig. 8A-B). We identified 2 significant L-R pairs (Vegfa-Vegfr1 and Vegfa-Vegfr2) in the VEGF signaling pathway. Notably, the Vegfa-Vegfr1 L-R pair mediated interactions from skeletal muscle cells to endothelial cells, while the Vegfa-Vegfr2 L-R pair facilitated signaling to myeloid cells (Figs. 8C-F, Supplemental Fig. 6). In the TENASCIN pathway, a moderate network of cell-cell interactions was identified (Supplemental Fig. 7A). Skeletal muscle cells exhibited robust activity as influencers and played moderate roles as senders and receivers, while endothelial cells demonstrated dominant roles as sender, receiver, mediator, and influencers (Supplemental Fig. 7B). We identified 1 significant L-R pair in the TENASCIN signaling pathway, comprising ligand gene Tnxb and receptor gene Sdc4 (Supplemental Fig. 7C). Interactions facilitated by the Tnxb-Sdc4 L-R pair revealed that skeletal muscle cells and endothelial cells communicate with each other and send outgoing signals to erythroid cells, mesenchymal stem cells, monocytes/macrophages, myeloid cells, and osteoblasts. These interactions within the TENASCIN signaling pathway support ECM remodeling, tissue repair, and adaptation to mechanical stress (Supplemental Fig. 7D).

**Figure 9:**
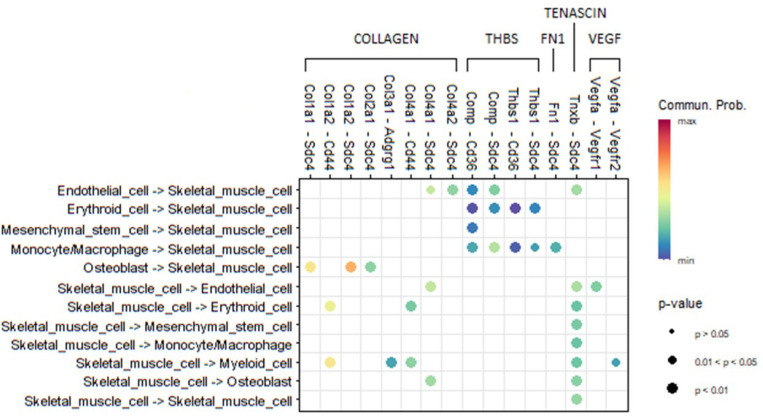
The comprehensive landscape of L-R pairs for skeletal muscle cells that either transmit signals to or receive signals from cells predominantly enriched in bone or bone marrow, potentially contributing to in bone-muscle crosstalk.

To further elucidate bone-muscle crosstalk, we generated the L-R pair landscape involved in bone and skeletal muscle interactions, as depicted in Fig. 9. Our analysis focuses on skeletal muscle cells that either transmit signals to or receive signals from cells mainly enriched in bone or bone marrow. Beyond the L-R pairs previously discussed, we identified additional interactions where skeletal muscle cells influence erythroid and myeloid cells via Col1a2-Cd44 and Col4a1-Cd44 through the COLLAGEN signaling pathway. Additionally, skeletal muscle cells modulate osteoblast activity through Col4a1-Sdc4, also mediated by the COLLAGEN signaling pathway. Our results provide a comprehensive map of L-R interactions in bone, skeletal muscle, and their crosstalk, highlighting the intricate communication networks mediated by key signaling pathways, such as COLLAGEN, THBS, FN1, VEGF, and TENASCIN, which play critical roles in tissue-specific functions, including ECM remodeling, hematopoietic niche regulation, immune signaling, and angiogenesis. The findings enhance our understanding of bone, skeletal muscle, and their crosstalk in maintaining musculoskeletal health, offering valuable insights for the development of therapeutic strategies targeting these pathways in musculoskeletal and metabolic disorders.

## Discussion

This study presents a detailed and comprehensive spatial and transcriptional characterization of cell-cell interactions within the bone and skeletal muscle microenvironments, highlighting the intricate intercellular communication networks that underpin tissue homeostasis, musculoskeletal crosstalk, and systemic metabolic regulation. By integrating ST with advanced computational deconvolution techniques, we systematically mapped L-R interactions across diverse cell types, offering novel insights into the molecular mechanisms driving musculoskeletal health and disease.

Our findings establish, for the first time, the feasibility of applying spatial transcriptomic analyses to bone and adjacent skeletal muscle tissues, overcoming longstanding technical challenges in preserving and analyzing these complex tissue types. Rigorous quality control metrics and distinct tissue-specific gene expression profiles underscore the robustness of our approach in delineating spatially resolved transcriptional landscapes. Successful identification of cortical bone, trabecular bone, bone marrow, and skeletal muscle regions, along with their distinct marker gene expression patterns, demonstrates the capability of ST to capture the spatial heterogeneity and transcriptional complexity of these tissues. In addition, the intercellular communication networks identified in this study revealed the intricate interplay among diverse cell populations, particularly osteoblasts, monocytes/macrophages, skeletal muscle cells, and endothelial cells. Notably, osteoblasts and monocytes/macrophages emerged as dominant players in their respective signaling pathways, reflecting their multifaceted roles in tissue remodeling, immune regulation, and ECM organization. Robust autocrine signaling observed in osteoblasts, erythroid cells, and monocytes/macrophages underscores the regulatory feedback mechanisms intrinsic to these microenvironments. For example, erythroid cells regulate their differentiation, survival, and function through intrinsic mechanisms, including transcription factors, autocrine signaling, and metabolic controls (65).

Furthermore, our findings provide compelling evidence for the spatially organized crosstalk between bone and skeletal muscle tissues. Signaling pathways such as the VEGF and TENASCIN in skeletal muscle cells, THBS and FN1 in monocytes/macrophages, alongside COLLAGEN and SPP1 in osteoblasts, represent key mediators of these communications. The identification of distinct outgoing and incoming signaling patterns highlights the functional specialization of cell types in coordinating bone-muscle interactions. For instance, skeletal muscle cells were prominent senders in the VEGF pathway, promoting angiogenesis and vascular remodeling (41, 63, 64), while osteoblasts exhibited strong incoming signals in the COLLAGEN and SPP1 pathways, supporting bone remodeling and hematopoietic niche regulation (48–52).

The analysis of L-R pairs in the key signaling pathways further provides novel insights into their functional roles. The COLLAGEN signaling pathway supports the structural integrity of bone, bone marrow, and skeletal muscle (48–52, 66–72). In bone, it provides tensile strength and regulates osteoblast and osteoclast activity (66, 69). In bone marrow, it forms the ECM, supporting hematopoietic stem cells and immune functions (48, 50, 70). In skeletal muscle, it scaffolds new fibers during repair (71,72). Osteoblasts were dominant in this pathway, with the Col1a2-Sdc4 pair mediating extensive interactions, emphasizing their central role in ECM organization and remodeling. The SPP1 signaling pathway is essential for bone remodeling, hematopoietic stem cell support, and muscle repair (73–75). Erythroid cells and monocytes/macrophages acted as receivers and mediators, while osteoblasts served as key senders via the Spp1-Cd44 L-R pair. Prominent autocrine signaling in osteoblasts, monocytes/macrophages, and erythroid cells highlighted their intra-population regulation. The THBS pathway plays a vital role in bone remodeling, stem cell niche maintenance, and muscle repair (56, 58, 76, 77). Monocytes/macrophages were key senders, while osteoblasts were dominant receivers. Among six L-R pairs, Comp-Sdc4 emerged as a critical mediator, facilitating robust interactions between monocytes/macrophages and multiple cell types, including endothelial and skeletal muscle cells. The FN1 pathway supports cell adhesion and tissue regeneration in bone, marrow, and muscle (62, 78–80). Monocytes/macrophages were prominent senders, transmitting signals to osteoblasts and other cell types via the Fn1-Sdc4 and Fn1-Cd44 pairs. Strong autocrine signaling reinforced their multifunctional role in ECM regulation. The VEGF pathway drives angiogenesis and vascular remodeling, essential for bone growth and muscle repair (81–85). Skeletal muscle cells acted as senders, signaling to endothelial cells via Vegfa-Vegfr1 and to myeloid cells via Vegfa-Vegfr2. Endothelial cells were dominant receivers, reflecting their central role in vascular niche maintenance. The TENASCIN pathway supports ECM remodeling, tissue repair, and bone-muscle crosstalk (40, 86–88). Skeletal muscle cells and endothelial cells exhibited moderate and strong signaling activity, respectively, with the Tnxb-Sdc4 pair mediating interactions across various cell types. These interactions facilitate adaptation to mechanical stress and promote tissue integrity.

The dysregulation of these signaling pathways is associated with a range of musculoskeletal and metabolic disorders, including osteoporosis, sarcopenia, and fibrosis (3–8). Our findings provide a valuable framework for targeting specific L-R interactions to restore tissue homeostasis and improve musculoskeletal health. For example, therapeutic modulation of the Col1a2-Sdc4 pair in the COLLAGEN pathway or the Comp-Sdc4 pair in the THBS pathway could offer novel strategies for enhancing bone and muscle regeneration. Future studies should focus on integrating additional spatial and temporal dimensions to further unravel the dynamics of these interactions. Incorporating other advanced imaging technologies, such as spatial proteomics, could provide complementary insights into the functional consequences of L-R interactions. Moreover, extending this approach to disease models and human tissues will be critical for translating these findings into clinical applications.

Despite its innovative approach and novel findings, this study may have several limitations that may serve as reference for future improved further studies. First, partially bone detachment during Visium slide preparation, due to the high density and mineralization of bone, may have affected spatial continuity and mapping accuracy, particularly at the bone-muscle interface. Second, the lack of single-cell resolution in Visium ST aggregates gene expression from multiple cells per spot, potentially obscuring individual cell contributions, despite computational deconvolution methods like SMART. Also, marker-based deconvolution may overrepresent dominant cell types, underestimating less abundant or poorly characterized populations. Third, the analysis provides only a static snapshot, without capturing temporal dynamics essential for understanding developmental and pathological processes. Moreover, the focus on healthy tissues excludes diseased states, aging, or injury, which might influence interaction networks. At last, but not the least, the absence of proteomic validation limits the interpretation of post-transcriptional dynamics, and the lack of comparisons with other spatial technologies leaves observed interactions unvalidated across platforms. Addressing these limitations in our future research will enhance the accuracy, resolution, and clinical relevance of spatially resolved analyses.

In conclusion, out study establishes a comprehensive map of spatially resolved cell-cell communication networks in femur and adjacent skeletal muscle tissues, advancing our understanding of the molecular basis of bone-muscle crosstalk. By identifying key signaling pathways and their functional implications, this work lays the foundation for novel therapeutic interventions aimed at preserving musculoskeletal health and mitigating related disorders.

## Materials and Methods

### Animal

A male C57BL/6 mouse was obtained from Charles River Laboratory (Wilmington, MA, USA). Prior to experimentation, the mouse was housed under pathogen-free conditions in an individually ventilated cage within the Animal Center at Tulane University. All handling procedures, including health monitoring and feeding, were conducted under sterile conditions. The mouse was provided ad libitum access to a standard cereal-based diet (Teklad 8604, Zeigler NIH-07, Purina 5001) and sterilized tap water. A 12-hour light/dark cycle was maintained throughout the study. All animal procedures complied with ethical guidelines and were approved by the Tulane University Institutional Animal Care and Use Committee (Approval # P0131).

### Tissue Harvest and Histology Preparation

The right femur and attached skeletal muscle were harvested immediately after sacrifice, bisected, and fixed in 10% buffered formalin at 4°C overnight. The sample were decalcified in 14% EDTA (pH 8) for two weeks on a shaker, with the solution refreshed every one to two days. Following decalcification, the sample were processed for paraffin embedding, and longitudinal 4 pm sections, including the femoral head, were obtained from the paraffin blocks.

RNA integrity was assessed using curls collected from the paraffin blocks. Sample achieving a DV200 threshold of ≥ 30% were considered suitable for further analyses. Spatial transcriptomic libraries were generated using the Visium CytAssist Spatial Gene Expression for FFPE Kit (10X Genomics) following the protocol outlined in Demonstrated Protocol CG000520. One tissue section was processed according to the Visium CytAssist Tissue Preparation Guide (Demonstrated Protocol CG000518). After deparaffinization, the section was stained with hematoxylin and eosin (H&E) and imaged at x20 magnification using a NanoZoomer S60v2MD scanner (Hamamatsu).

After imaging, slide was decoverslipped, followed by hematoxylin destaining, decrosslinking, and hybridization using the Visium Mouse Transcriptome Probe Set v1.0, targeting 20,551 mouse genes. Hybridized and ligated probes were released from the tissue via permeabilization and captured on Visium Slide oligos. The barcoded ligation products were amplified and indexed. Libraries were sequenced on an Illumina NextSeq 2000 platform with 2 × 96 bp paired-end sequencing, achieving a depth exceeding 100 million reads per sample.

### Visium Data Processing and Analysis

Demultiplexing and sequence alignment were conducted using 10x Genomics Space Ranger (v2.1.1), coupled with registration to H&E-stained images. A feature matrix in h5 format was subsequently imported into Seurat (v5.1.0). We applied a feature count filter to remove features present at levels less than 1.0 in at least 80% of cells. Normalization was performed using SCTransform (Seurat). Following normalization, unsupervised graph-based clustering was executed utilizing the Louvain clustering algorithm with a resolution parameter of 0.5, post-dimensionality reduction via principal component analysis. Biomarkers for each cluster were identified, setting a positive fold change threshold of 1.5.

### Differential Gene Expression and Functional Enrichment Analysis

Differentially expressed genes between skeletal muscle cell cluster and other cell clusters was detected using the R package DEsingle (v1.18.0) (89). The significance criteria were as follows: adjusted *p*-value < 0.05, absolute log2 fold change > 1. Functional enrichment of a set of genes was conducted using CluoGO (v2.5.9) (90) under default settings. A Bonferroni-corrected *p*-value threshold of < 0.05 was employed to determine statistical significance.

### Cell Type Deconvolution Analysis

Cell-type-specific gene expression and spatial composition were inferred using the SMART v1.0 computational framework, a marker-gene-assisted deconvolution method based on semi-supervised topic models (32). SMART outperforms some of the best-performing reference-based and reference-free methods (91–93) for ST data when an ideal reference dataset is unavailable (32). Marker genes were used to decompose mixed gene expression profiles at each spatial spot using constrained optimization algorithms. SMART calculated the proportion of each cell type in every spatial spot, annotated dominant cell-type spots, and assigned spatially resolved cell-type-specific gene expression. This approach improves spatial resolution and enhances biological interpretability, enabling characterization of tissue architecture and microenvironments. In this study, we curated marker genes from the Mouse Cell Atlas (34) and the CellMarker 2.0 database (35), ensuring comprehensive and biologically relevant reference datasets. For each spatial spot, the dominant cell type was annotated based on the highest proportion of each cell type.

### Intercellular Communication Analysis

CellChat v2.0 (33) was employed to infer spatially proximal cell-cell communication from the spatial transcriptomic data using default settings. Intercellular interaction analysis was performed to calculate the number of interactions between cell types and their corresponding L-R pairs. Additionally, CellChat assessed the communication dynamics between cell clusters in terms of signaling pathways. This analysis identified overexpressed receptors and ligands within each cell cluster, quantifying the interaction strength between clusters through calculated communication probabilities. Specifically, the outgoing (sender) and incoming (receiver) signaling patterns were identified by detecting ligand or receptor expression in specific cell types, quantifying communication strength using probabilistic modeling, and applying non-negative matrix factorization to extract dominant signaling patterns. For identification of key signaling pathways, CellChat groups L-R pairs into known signaling pathways and ranks pathways based on their global communication strength and importance in network structure. Significant interactions were determined using a permutation test, applying a significance threshold of *p* < 0.05. The permutation test involved random shuffling of cell-type labels, followed by recalculating communication probabilities to evaluate the statistical significance of the interactions.

## Supplementary Material

This is a list of supplementary files associated with this preprint. Click to download.

- SupplementalFigures.docx

## Figures and Tables

**Table 1:** 10X Genomics Visium Transcriptomics Data Summary

Spots	Number
Number of Spots under Tissue	2,660
Mean Reads per Spot	105,186
Median UMI Counts per Spot	818
Median Genes per Spot	506
Genes Detected	19,199

**Table 2:** Enrichment Analysis of Differentially Expressed Genes Identified Through Comparative Analysis between Bone and Skeletal Muscle Tissues

Enriched GO Terms	p-value
Striated Muscle Contraction	3.98E-08
Collagen chain trimerization	1.92E-05
Defective VWF binding to collagen type I	5.38E-05
Collagen degradation	1.82E-04
Assembly of collagen fibrils and other multimeric structures	1.90E-04
Enhanced binding of GP1BA variant to VWF multimer:collagen	2.90E-04
Defective binding of VWF variant to GPIb:IX:V	2.90E-04
Defects of platelet adhesion to exposed collagen	3.79E-04
Non-integrin membrane-ECM interactions	6.78E-04
MET activates PTK2 signaling	9.50E-04
ECM proteoglycans	0.00119712
Anchoring fibril formation	0.001309816
Degradation of the extracellular matrix	0.001365036
Apoptosis induced DNA fragmentation	0.001674796
RUNX3 Regulates Immune Response and Cell Migration	0.002082612
Collagen biosynthesis and modifying enzymes	0.003195066
Crosslinking of collagen fibrils	0.003285646
Collagen formation	0.006642439
Scavenging by Class A Receptors	0.012947068
DNA Damage Recognition in GG-NER	0.017778019
RUNX2 regulates osteoblast differentiation	0.017946737
NCAM1 interactions	0.025878509
Integrin cell surface interactions	0.02810591
RUX2 regulates bone development	0.029271148
